# Assessing the Use of the sGC Stimulator BAY-747, as a Potential Treatment for Duchenne Muscular Dystrophy

**DOI:** 10.3390/ijms22158016

**Published:** 2021-07-27

**Authors:** Shalini Murali Krishnan, Johannes Nordlohne, Lisa Dietz, Alexandros Vakalopoulos, Petra Haning, Elke Hartmann, Roland Seifert, Jörg Hüser, Ilka Mathar, Peter Sandner

**Affiliations:** 1 Bayer AG, R&D Pharmaceuticals, Pharma Research Center, 42113 Wuppertal, Germany; shalini.mkrishnan@gmail.com (S.M.K.); johannes.nordlohne@bayer.com (J.N.); lisa.dietz@bayer.com (L.D.); alexandros.vakalopoulos@bayer.com (A.V.); petra.haning@bayer.com (P.H.); hartmann06@arcor.de (E.H.); joerg.hueser@bayer.com (J.H.); ilka.mathar@bayer.com (I.M.); 2 Institute of Pharmacology, Hannover Medical School, 30625 Hannover, Germany; Seifert.Roland@mh-hannover.de

**Keywords:** duchenne muscular dystrophy, sGC stimulator, mdx/mTR^G2^ mice, skeletal muscle function, skeletal muscle damage, inflammation, fibrosis

## Abstract

Duchenne muscular dystrophy (DMD) is a severe and progressive muscle wasting disorder, affecting one in 3500 to 5000 boys worldwide. The NO-sGC-cGMP pathway plays an important role in skeletal muscle function, primarily by improving blood flow and oxygen supply to the muscles during exercise. In fact, PDE5 inhibitors have previously been investigated as a potential therapy for DMD, however, a large-scale Phase III clinical trial did not meet its primary endpoint. Since the efficacy of PDE5i is dependent on sufficient endogenous NO production, which might be impaired in DMD, we investigated if NO-independent sGC stimulators, could have therapeutic benefits in a mouse model of DMD. Male mdx/mTR^G2^ mice aged six weeks were given food supplemented with the sGC stimulator, BAY-747 (150 mg/kg of food) or food alone (untreated) ad libitum for 16 weeks. Untreated C57BL6/J mice were used as wild type (WT) controls. Assessments of the four-limb hang, grip strength, running wheel and serum creatine kinase (CK) levels showed that mdx/mTR^G2^ mice had significantly reduced skeletal muscle function and severe muscle damage compared to WT mice. Treatment with BAY-747 improved grip strength and running speed, and these mice also had reduced CK levels compared to untreated mdx/mTR^G2^ mice. We also observed increased inflammation and fibrosis in the skeletal muscle of mdx/mTR^G2^ mice compared to WT. While gene expression of pro-inflammatory cytokines and some pro-fibrotic markers in the skeletal muscle was reduced following BAY-747 treatment, there was no reduction in infiltration of myeloid immune cells nor collagen deposition. In conclusion, treatment with BAY-747 significantly improves several functional and pathological parameters of the skeletal muscle in mdx/mTR^G2^ mice. However, the effect size was moderate and therefore, more studies are needed to fully understand the potential treatment benefit of sGC stimulators in DMD.

## 1. Introduction

Duchenne muscular dystrophy (DMD) is an X-linked recessive disorder caused by mutations in the gene encoding the dystrophin protein. The lack of a fully functional dystrophin protein results in severe and progressive weakness of both the skeletal and cardiac muscles. Although it is considered a rare disease, DMD is the most common form of muscular dystrophy affecting one in 3500 to 5000 boys worldwide [1] with these boys generally diagnosed between the age of 3 to 5 years. The disease primarily affects the pelvic-girdle muscles at the initial stages, therefore as adolescents the boys are dependent on assisted mobility devices such as crutches or a wheelchair. As the disease progresses, in young adults affected with DMD, muscle dysfunction starts to affect cardiac and respiratory function, eventually requiring artificial ventilation to aid respiration. Most patients do not survive beyond 25–30 years of age, with cardiac failure being the major cause of death. In order to improve the quality and longevity of life, currently a comprehensive multi-disciplinary approach is used to manage DMD, involving pharmacological and non-pharmacological therapies that mostly improve symptoms, along with regular assessments of orthopedics, cardiology, pulmonology, osteology as well as mental health [2,3,4].

In the recent years, DMD has been an attractive indication for gene-based therapies. There has been growing interest especially with therapies based on ‘exon skipping’ approaches, including Exondys51, Vyondys53 and Viltolarsen approved in the last year [5]. While there are several studies currently in clinical trials aiming to restore dystrophin production, thereby treating the underlying cause of DMD, currently, most therapies aid by treating the symptoms [5]. To date, the only symptom-modifying therapy approved for DMD are glucocorticoids, including prednisone and deflazacort, which improve skeletal muscle strength and function, whereas other therapies are used off-label to manage respiratory and cardiovascular symptoms. While gene therapies restoring expression of functional dystrophin in skeletal muscle and heart would ideally provide a cure for DMD, available therapies are effective only in a selected subpopulation of DMD patients, and novel improved gene therapies still need to prove efficacy. Moreover, while future therapies might be able to greatly reduce mortality, residual loss of muscle cells is likely to translate into chronic and progressive morbidity requiring further medical treatment. 

The dystrophin glycoprotein complex (DGC), present in the skeletal and cardiac muscle forms a link between the intracellular cytoskeleton and extracellular matrix and plays a key role in membrane sarcolemma stability, protecting the muscle during repeated contractions. DGC also serves as a scaffold to assemble a macromolecular complex with numerous, yet ill-defined functions. One of the subcomplexes linked to the DGC via the α-syntrophin protein is neuronal NO synthase (nNOS) [6,7]. The absence of functional dystrophin protein also displaces nNOS from the membrane, resulting in impaired skeletal muscle perfusion and exercise efficiency in mdx mice as well as in DMD boys [8]. This occurs directly as a consequence of impaired NO synthesis and cGMP production in the skeletal muscle. Therefore, one treatment approach to improve exercise efficiency could target the low endogenous cGMP production.

The nitric oxide-soluble guanylyl cyclase–cyclic guanosine monophosphate–phosphodiesterase (NO-sGC-cGMP-PDE) pathway is known to play an important role in skeletal muscle function and exercise. During exercise, in order to meet the metabolic demands of the skeletal muscle, there is increased dilatation and perfusion of the blood vessels in the skeletal muscles, described as functional sympatholysis. NO improves functional sympatholysis by stimulating the sGC enzyme, triggering cGMP production and causing vasodilatation and perfusion of the skeletal muscles, which in turn increases oxygen delivery to the working skeletal muscles and improves exercise efficiency [7,9]. In addition to perfusion matching via functional sympatholysis, the NO-sGC-cGMP axis has been suggested to modulate muscle microtubule organization and mitochondrial oxidative phosphorylation ultimately regulating muscle fatigability [7]. 

Therefore, cGMP increasing pharmacological mechanisms came into focus for treating muscular dystrophies. Previously, PDE5 inhibitors, which elevate cGMP levels by preventing its breakdown, have been assessed as a potential therapeutic for the treatment of DMD. Preclinical studies showed that administration of PDE5 inhibitors such as sildenafil or tadalafil reduced muscle damage [10], reversed cardiac dysfunction and delayed the onset of cardiomyopathy [11,12], improved diaphragm contractility and reduced fibrosis [13] in mdx mice. In a small-scale clinical trial with 10 DMD patients who displayed functional ischemia despite being on corticosteroids, treatment with sildenafil or tadalafil enhanced blood flow and improved exercise-induced functional sympatholysis in a dose-dependent manner in the skeletal muscle [14]. In addition, tadalafil was able to alleviate muscle ischemia in eight out of nine patients with Becker muscular dystrophy (BMD), a milder form of muscular dystrophy which is also characterized by dystrophin mutations [15]. Despite these positive outcomes in smaller pilot trials, a large-scale Phase III trial assessing the use of tadalafil for the treatment of DMD did not reach its primary endpoint of improving six-minute walking distance after 48 weeks of treatment [16]. While tadalafil also showed no signals in a comprehensive ambulatory assessment used as secondary endpoint, it was interesting to note that in a subgroup analysis performed on a prespecified subgroup of boys over 10 years of age, the total and shoulder-level upper limb scores showed lesser decline following treatment with tadalafil compared to placebo treatment [16]. This suggests that PDE5 inhibitors could show less decline in ambulation in younger and less disabled DMD boys [6], and in addition, it also suggests that enhancement of cGMP with PDE5 inhibitors was beneficial but still under the critical threshold to show clinical benefit. 

The failure or low efficacy of PDE5 inhibitors in DMD patients could have been due to the reason that the PDE5 inhibitors rely on NO and the downstream cGMP production in order to effectively work by inhibiting the breakdown of cGMP. In the early 2000s, a new class of cGMP enhancing compounds, known as sGC stimulators were discovered [17,18]. These compounds target the sGC enzyme and can actively cause cGMP production without requiring endogenous NO but also synergistically potentiate the effects of NO, even if the levels are very low [17]. Therefore, in this study we examined the therapeutic potential of the sGC stimulator, BAY-747, in vivo in a mouse model of DMD (mdx/mTR^G2^ mice). BAY-747 (*N*-(2-amino-2-methylbutyl)-8-[(2,6-difluorobenzyl)oxy]-2,6-dimethylimidazo [1,2-a]pyridine-3-carboxamide) belongs to a new chemical class of long-acting sGC stimulators and shares its molecular mode of action with riociguat and vericiguat [19,20]. Treatment with BAY-747 improved skeletal muscle function, reduced serum creatine kinase levels, and biomarkers of inflammation and fibrosis in the skeletal muscle with no effect on myeloid cell infiltration and collagen accumulation. Therefore, findings from this study suggest that sGC stimulators might have treatment potential in muscular dystrophies.

## 2. Results

### 2.1. The NO-sGC-PDE Pathway Is Largely Downregulated in the Skeletal Muscle of Mdx/mTR^G2^ Mice

**Figure 1:**
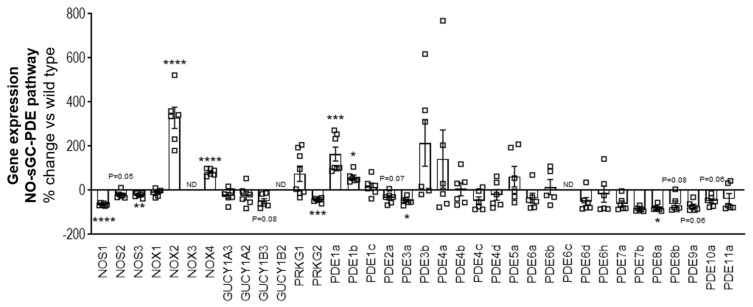
The NO-sGC-PDE pathway is largely downregulated in the skeletal muscle of mdx/mTR^G2^ mice. Fold change of mRNA expression of various genes in the NO-sGC-PDE signaling pathway in 22-week old mdx/mTR^G2^ versus wild type C57BL6/J mice in the quadriceps. All values are expressed as mean ± SEM. * *p* < 0.05; ** *p* < 0.01; *** *p* < 0.001; **** *p* < 0.0001; ns, not significant for unpaired *t*-test.

We measured gene expression at the mRNA level of genes involved in regulation of the NO-sGC-PDE pathway including the isoforms of NOS (nitric oxide synthase), NOX (NADPH oxidase), PDE (phosphodiesterase) and subunits of sGC (soluble guanylate cyclase). It was observed that important components of the NO-sGC-PDE like the NO producing eNOS and iNOS, the beta-1 subunit of the NO-receptor sGC as the cGMP downstream target PRKG2 were downregulated in the quadriceps of 22-week-old adult mdx/mTR^G2^ mice compared to age-matched wild type mice (Figure 1). In addition, NOX2 which can enhance ROS-formation which can lead to decoupling of the NO-signaling was upregulated. We were not able to detect NOX3, GUCY1B2 and PDE6c gene expression in the quadriceps muscle of these mice.

### 2.2. BAY-747 Administration Increased Cgmp Levels In Skeletal Muscles of Mdx/Mtr^g2^ Mice

**Figure 2:**
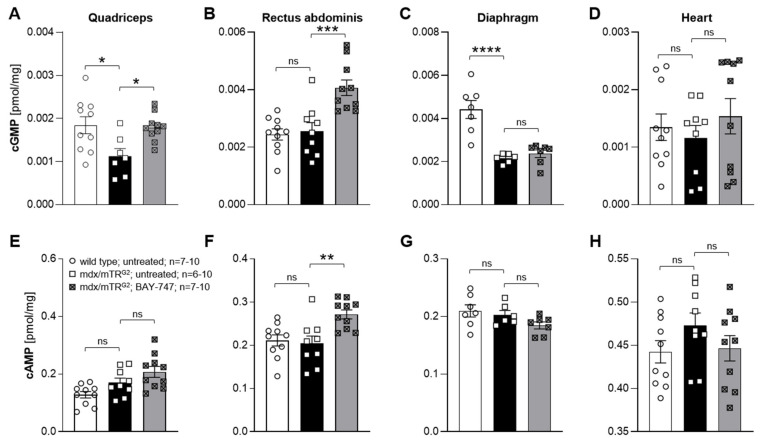
BAY-747 administration increased cGMP levels in skeletal muscles of mdx/mTR^G2^ mice. cGMP (**A**–**D**) and cAMP (**E**–**H**) levels were measured in various skeletal muscles including (**A**,**E**) quadriceps, (**B**,**F**) rectus abdominis, (**C**,**G**) diaphragm as well as (**D**,**H**) the heart, respectively. * *p* < 0.05; ** *p* < 0.01; *** *p* < 0.001; **** *p* < 0.0001; ns, not significant for one-way ANOVA followed by Dunnett’s multiple comparisons post-hoc test.

Previous studies have shown that skeletal muscles in DMD patients and mdx mice have low endogenous cGMP production [10,11,13,21,22]. sGC stimulators work via inducing cGMP production, independent of nitric oxide. Therefore, we examined if endogenous cGMP levels in various skeletal muscles of mdx/mTR^G2^ mice were dysregulated, and if treatment with BAY-747 was able to restore these levels (Figure 2A–D). It was observed that compared to wild type mice, the cGMP levels were reduced in the quadriceps and diaphragm skeletal muscles of mdx/mTR^G2^ mice (0.001125 ± 0.0001757 and 0.0002151 ± 0.00008851 pmol/mg in mdx/mTR^G2^ mice versus 0.001842 ± 0.0001969 and 0.0004417 ± 0.0004149 pmol/mg in wild type mice, respectively, *p* < 0.05). Treatment with BAY-747 increases cGMP by a significant level in the quadriceps and rectus abdominis muscles (0.001794 ± 0.0001006 and 0.004068 ± 0.0002696 versus 0.001125 ± 0.0001757 and 0.002558 ± 0.0002967 pmol/mg in untreated mdx/mTR^G2^ mice, respectively, *p* < 0.05). However, a significant increase was not observed in the diaphragm muscle. cGMP levels in the cardiac muscle were similar in all groups. cAMP levels, on the other hand, were approximately 100-fold higher in the skeletal muscles assessed and 450-fold in the cardiac muscle compared to cGMP levels in the respective tissue. The levels of cAMP in both the skeletal and cardiac muscles were similar in both wild type and untreated mdx/mTR^G2^ mice (Figure 2E–H), with BAY-747 causing a significant increase in cAMP levels only in the rectus abdominis (0.2715 ± 0.01037 versus 0.2039 ± 0.01761 pmol/mg, *p* < 0.05). 

BAY-747 treatment improved forelimb grip strength but not the four-limb hang in mdx/mTR^G2^ mice.

**Figure 3:**
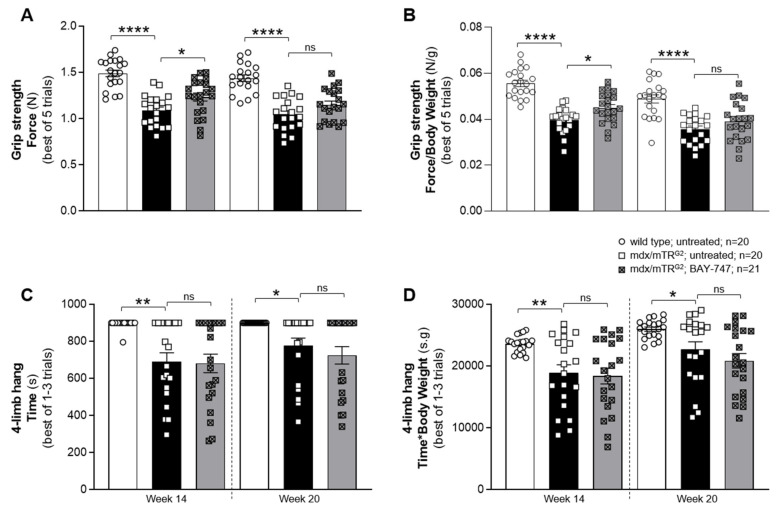
BAY-747 treatment improved forelimb grip strength but not the four-limb hang capacity in mdx/mTR^G2^ mice. Treatment with BAY-747 improves forelimb grip strength capacity (**A**,**B**) but does not improve four-limb hang capacity (**C**,**D**) of mdx/mTR^G2^ mice compared to untreated mdx/mTR^G2^ mice at the interim time point (Week 14) but not to a significant level at the final time point assessed (Week 20). * *p* < 0.05; ** *p* < 0.01; **** *p* < 0.0001; ns, not significant for one-way ANOVA followed by Dunnett’s multiple comparison post-hoc test.

Treatment with BAY-747 was able to increase cGMP in some but not all the skeletal muscles examined, therefore it was important to assess whether this resulted in improved functional capacity of skeletal muscles in mdx/mTR^G2^ mice. To do this, we performed the four-limb hang and grip strength assessments first at 8 weeks (interim time point) and then at 16 weeks (final time point) post commencement of BAY-747 treatment. With the forelimb grip strength assessment, it was evident that mdx/mTR^G2^ mice have impaired strength in the forelimbs already at age 14 weeks (1.089 ± 0.03670 N versus 1.49 ± 0.03609 N for age-matched wild type mice, *p* < 0.05), as well as 20 weeks (1.046 ± 0.03918 N versus 1.438 ± 0.03586 N for age-matched wild type mice, *p* < 0.05) (Figure 3A). Treatment with BAY-747 showed an improvement in forelimb grip strength 8 weeks post-treatment (1.234 ± 0.04339 N versus 1.089 ± 0.03670 N for untreated mdx/mTR^G2^ mice, *p* < 0.05). However, this treatment effect lost significance 16 weeks post-treatment (1.151 ± 0.03909 N versus 1.046 ± 0.03918 N for untreated mdx/mTR^G2^ mice, *p* > 0.05) (Figure 3A).

Similar to the forelimb grip strength assessment, the four-limb hang assessment also showed that the mdx/mTR^G2^ mice had reduced skeletal muscle function as these mice were not able to hang on for as long as the wild type mice (691.4 ± 47.27 s versus 894.8 ± 5.25 s, *p* < 0.05 at 14 weeks of age and 776.8 ± 40.9 s versus 900.0 ± 0.00 s, *p* < 0.05 at 20 weeks) (Figure 3C). In contrast to the improvement seen with the grip strength measurement at 14 weeks, we observed no significant improvement with the four-limb hang following treatment with BAY-747 in the mdx/mTR^G2^ mice at both time points assessed. Furthermore, since body weight is likely to impact skeletal muscle functional capacity, we also analyzed these results by normalizing to body weight (Figure 3B,D). We found that body weight appeared to have no significant effect on the performance of these assessments.

### 2.3. Treatment with BAY-747 Improved Speed but Not the Overall Distance Run by Mdx/Mtr^g2^ Mice in the Running Wheel Functional Assessment

**Figure 4:**
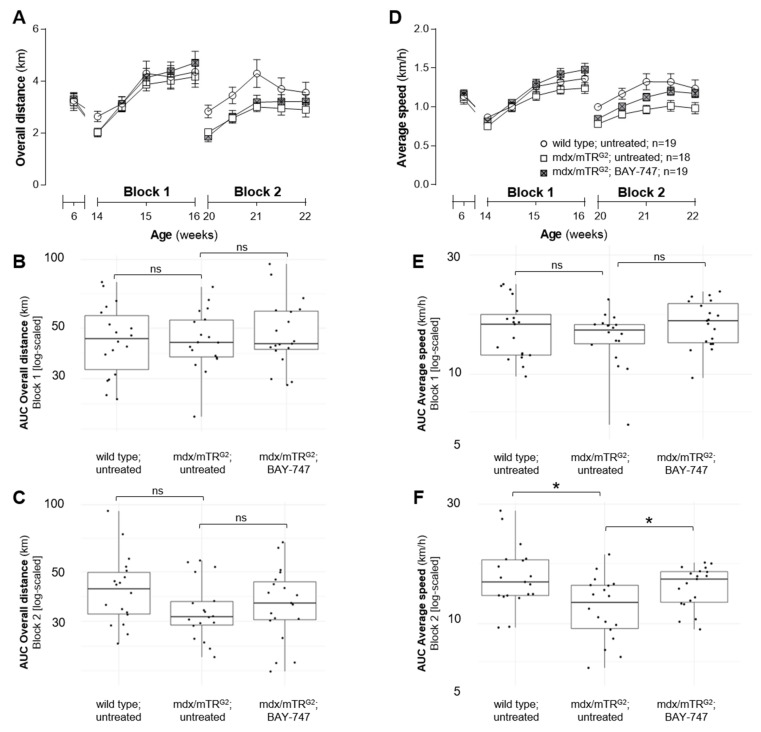
Treatment with BAY-747 improved speed but not the overall distance run by mdx/mTR^G2^ mice in the running wheel functional assessment. In the voluntary running wheel assessment, mice treated with BAY-747 run the same overall distance (**A**) but at a faster average speed (**D**) than untreated mdx/mTR^G2^ mice. AUC was calculated for each mouse and log-transformed for Blocks 1 and 2 for overall distance (**B**,**C**) and average speed (**E**,**F**), respectively. * *p* < 0.05; ns, not significant. For statistical analysis method, please refer to the Materials and Methods section.

The voluntary running wheel is another method used to assess skeletal muscle function, however unlike the grip strength and four-limb hang methods, the running wheel does not involve handling of the mice immediately prior to collection of data. At first, mice were placed in individual running wheel cages for 1 week and overall distance was measured, based on which the mdx/mTR^G2^ mice were randomized into either receiving untreated- or BAY-747-supplemented food. At this age, no significant difference was observed between all three groups for the overall distance or average speed run (Figure 4A,D). Between ages 14 and 16 weeks (Block 1), all three groups show a progressive increase in both the overall distance run from around 2.017 to 4.708 km as well as average speed from around 0.7503 to 1.4788 km/h (Figure 4A,D). The slope of increase appears to be more evident between age 14–15 weeks, during which a significant difference was observed between wild type and untreated mdx/mTR^G2^ mice in the first few days (Figure 4A,D). Between ages 15 to 16 weeks, no difference was observed between either of the groups in the overall distance run (Figure 4A). During this time, the average speed was also similar between wild type and untreated mdx/mTR^G2^ mice. However, the mdx/mTR^G2^ mice on BAY-747 food were able to run significantly faster than untreated wild type and mdx/mTR^G2^ mice (Figure 4D,F). 

Interestingly, with the progression of age and the disease, the results observed between ages 20 and 22 weeks (Block 2) were quite different from that of Block 1. Firstly, the decreased ability of skeletal muscle function in the untreated mdx/mTR^G2^ mice compared to wild type mice was more pronounced in both the overall distance run as well as the average speed (Figure 4A,C,D,F). Furthermore, while there was no difference in the overall distance between mdx/mTR^G2^ mice on untreated versus BAY-747-supplemented food, the mice treated with BAY-747 were able to run this distance at a much faster pace compared to the untreated mdx/mTR^G2^ mice.

### 2.4. BAY-747 Treatment Reduced Serum Creatinine Kinase but Did Not Improve Skeletal Muscle Pathology of mdx/mTR^G2^ Mice

**Figure 5:**
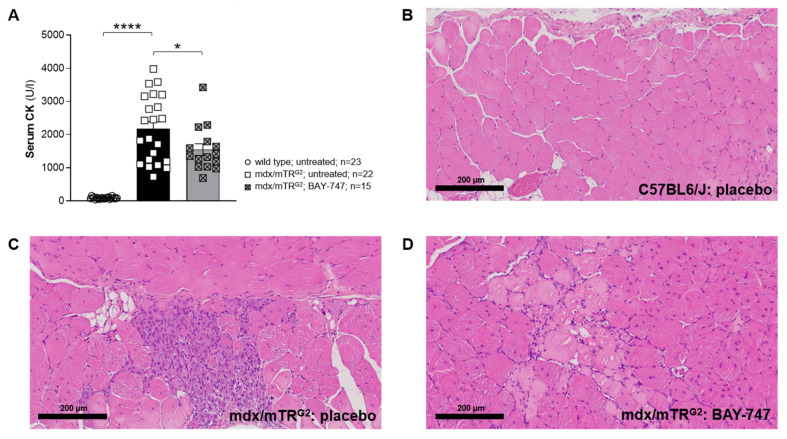
BAY-747 treatment reduced serum creatinine kinase but did not improve skeletal muscle pathology of mdx/mTR^G2^ mice. The 16-weeks of treatment with BAY-747 reduces circulating creatine kinase levels (**A**) in the mdx/mTR^G2^ mice compared to untreated mice. However, BAY-747 treatment for 16 weeks did not improve skeletal muscle pathology in mdx/mTR^G2^ mice. Representative images of H&E-stained rectus abdominis tissue sections of (**B**) wild type: untreated; (**C**) mdx/mTR^G2^: untreated; and (**D**) mdx/mTR^G2^: BAY-747. * *p* < 0.05; **** *p* < 0.0001; ns, not significant for one-way ANOVA followed by Dunnett’s multiple comparison post-hoc test.

Serum creatine kinase (CK) is a key biomarker of skeletal muscle damage, and in patients with BMD and DMD it has been found that the levels of CK are up by 25- to 200-fold [23] compared to healthy individuals. At 22 weeks of age, we observed that mdx/mTR^G2^ mice have a nearly 25-fold increase (2178 ± 214.2 U/L) in serum CK levels compared to wild-type mice (91.26 ± 6.25 U/L, *p* < 0.05). Following 16 weeks of treatment with BAY-747, the levels were significantly reduced to 1551 ± 176.1 U/L (*p* < 0.05 versus untreated mdx/mTR^G2^) (Figure 5A). Furthermore, although we observed that BAY-747 treatment was able to reduce serum CK levels, assessment of skeletal muscle pathology for degeneration and necrosis showed no significant improvement in the mdx/mTR^G2^ mice (Figure 5C,D). Wild type mice showed no signs of degeneration or necrosis and were given a score of 0 (i.e., no finding present; Figure 5B). Skeletal muscle injury was characterized by focal or multifocal acute necrosis of muscle fibers or older degenerative/atrophic lesions with signs of regeneration. In acute necrotic lesions, injured fibers were pale eosinophilic with loss of striation. The fibers appeared swollen with nuclear debris at the periphery along the sarcolemma (Figure 5D). In older lesions (Figure 5C) degeneration/regeneration is reflected by a more basophilic appearance of affected fibers, centrally located nuclei and a decreased fiber diameter (atrophy). Besides the age of the lesions, the area of damage is similar in untreated and BAY-747-treated mdx/mTR^G2^ mice (Grade 3—moderate). Focal lesions of different age can be found in one animal side by side.

### 2.5. Treating mdx/mTR^G2^ Mice with BAY-747 Reduced Expression of Markers of Inflammation at the Gene Level but Not Immune Cell Infiltration into the Skeletal Muscle

**Figure 6:**
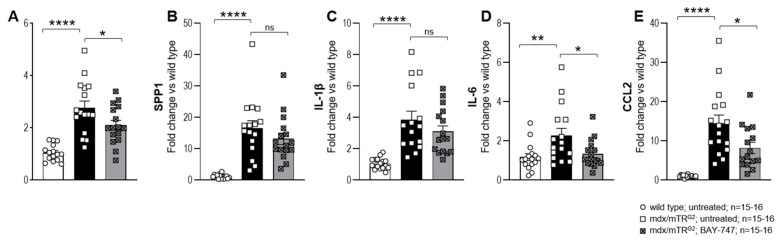

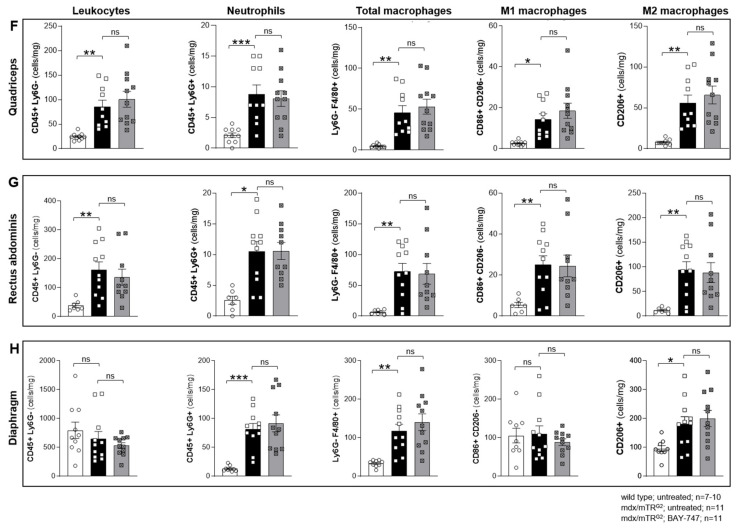
Treatment with BAY-747 on mdx/mTR^G2^ mice reduces expression of markers of inflammation at the gene level but not immune cell infiltration into the skeletal muscle. The effect of treatment with BAY-747 on levels of mRNA expression on pro-inflammatory genes (**A**) TNF, (**B**) SPP1, (**C**) IL-1β, (**D**) IL-6 and (**E**) CCL2 in the quadriceps and immune cell infiltration of (**F**) quadriceps, (**G**) rectus abdominis and (**H**) diaphragm of mdx/mTR^G2^ mice. * *p* < 0.05; ** *p* < 0.01; *** *p* < 0.001; **** *p* < 0.0001; ns, not significant for one-way ANOVA followed by Dunnett’s multiple comparison post-hoc test.

Markers of inflammation, including that of pro-inflammatory cytokines such as tumor necrosis factor (TNF), osteopontin (SPP1), interleukin (IL)-1β and IL-6 and the chemokine CCL2 were all significantly upregulated in the quadriceps of mdx/mTR^G2^ mice at the mRNA level (Figure 6A–E), and this translated to an increase in infiltration of immune cells including that of leukocytes, neutrophils and macrophages into various skeletal muscles (Figure 6F–H). A total 16 weeks of treatment with BAY-747 was able to reduce the expression of some of these markers including TNF, IL-6 and CCL2 (Figure 6A,D,E), however the decrease in expression of SPP1 and IL-1β was not statistically significant (Figure 6B,C). Although we saw a decrease in some of the markers of inflammation at the gene expression level, this appeared to have no impact on immune cell infiltration into various skeletal muscles at least at 16-weeks post-treatment with BAY-747 (Figure 6F–H). 

### 2.6. Treatment with BAY-747 Reduces Expression of Markers of Fibrosis at the Gene Level but Not Collagen Accumulation in the Skeletal Muscle of mdx/mTR^G2^ Mice

**Figure 7:**
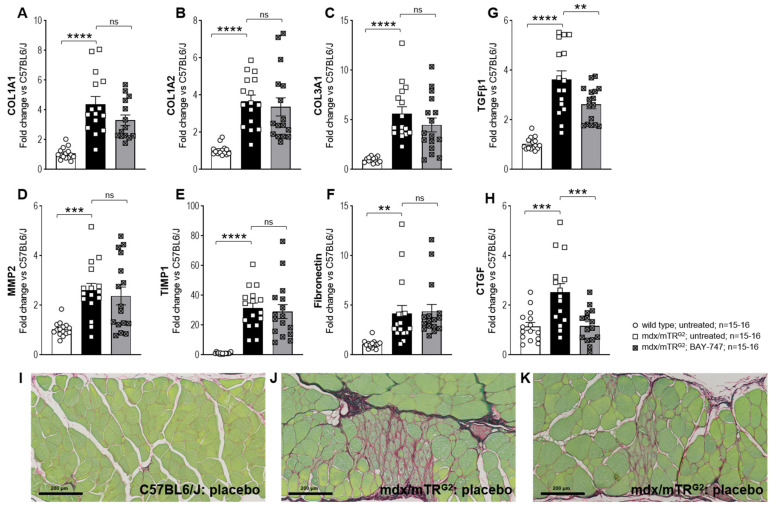
Treatment with BAY-747 reduces expression of markers of fibrosis at the gene level but not collagen accumulation in the skeletal muscle of mdx/mTR^G2^ mice. The effect of treatment with BAY-747 on levels of mRNA expression on pro-fibrotic genes (**A**) COL1A1, (**B**) COL1A2, (**C**) COL3A1, (**D**) MM2, (**E**) TIMP1, (**F**) fibronectin, (**G**) TGFβ1, (**H**) CTGF in the quadriceps. Representative images of Sirius Red/Fast Green-stained rectus abdominis tissue sections of (**I**) wild type: untreated; (**J**) mdx/mTR^G2^: untreated; and (**K**) mdx/mTR^G2^: BAY-747. ** *p* < 0.01; *** *p* < 0.001; **** *p* < 0.0001; ns, not significant for one-way ANOVA followed by Dunnett’s multiple comparison post-hoc test.

Similar to pro-inflammatory markers, mRNA expression of markers of fibrosis including that of various collagen subtypes (COL1A1, COL1A2, COL3A1), matrix metalloproteinase 2 (MMP2), tissue inhibitor of matrix metalloproteinase 1 (TIMP1), fibronectin, transforming growth factor β (TGFβ) and connective tissue growth factor (CTGF) were all significantly increased in the quadriceps muscle of 22-week old mdx/mTR^G2^ mice compared to age-matched wild type mice (Figure 7A–H). Only genes involved in the TGFβ-dependent signaling pathway such as TGFβ and CTGF were significantly reduced following treatment with BAY-747 (Figure 7G,H). However, staining with Sirius Red Fast Green showed no reduction in collagen accumulation in various skeletal muscle tissues following treatment with BAY-747 as compared to untreated mdx/mTR^G2^ mice (Figure 7I–K). In wild type mice no collagen deposition was observed (Grade 0; Figure 7I), whereas in the mdx/mTR^G2^ mice (Figure 7J,K), the collagen stain displays representative cases of slight (Grade 2) fibrosis. Severity score assessed for both untreated and BAY-747-treated mdx/mTR^G2^ mice were comparable.

## 3. Supplementary Results

### 3.1. Body Weight and Food Consumption

Since the substance was administered in the food, body weight and food consumption were monitored weekly. The body weight of mice increased over time in all groups (Supplementary Figure S1A), however no significant difference in weight gain was observed between groups (29.19 ± 0.396 g for untreated wild type; 29.84 ± 0.658 g for untreated mdx/mTR^G2^; 30.17 ± 0.837 g for mdx/mTR^G2^ BAY-747 at 22 weeks of age). Food consumption between all groups was relatively stable throughout the treatment period (Supplementary Figure S1B). There was no difference observed in the amount of food consumed by the mdxmTR^G2^ mice irrespective of whether it was untreated food (35.52 ± 1.035 g) or BAY-747-supplemented food (35.86 ± 0.917 g). The peaks observed at certain time points was due to the top of fresh food the week prior, during which the mice generally tend to consume more food. Since this was observed in the first cohort of mice, in the following cohort the food was topped up weekly to obtain steady food consumption. 

### 3.2. BAY-747 Has a Dose-Dependent Blood-Pressure Reducing (Pharmacodynamic) Effect

Prior data with other sGC stimulators demonstrated a dose-dependent blood-pressure reducing effect [19,24]. Therefore, in a small cohort of mice, radiotelemetry was used to identify an appropriate dose of BAY-747 by observing the pharmacodynamic effects (i.e., on blood pressure and heart rate) by administering food supplemented with either untreated, BAY-747 at 50 or 150 mg/kg of food. Administration of BAY-747 showed a dose-dependent reduction in systolic blood pressure (Supplementary Figure S2A). The reduction is systolic blood pressure was accompanied by a compensatory increase in heart rate (Supplementary Figure S2B), and this was more evident in the night-time (active) period compared to the daytime (inactive period). These hemodynamic effects were mild with 50 mg/kg of BAY-747 but more evident (mild to moderate) with 150 mg/kg of BAY-747, compared to untreated food in both wild type and mdx/mTR^G2^ mice. There was no major impact on diastolic blood pressure and only minimal impact on mean arterial pressure at both 50 and 150 mg/kg of BAY-747 compared to untreated food (Supplementary Figure S2C,D). Therefore, based on this data, all experiments in this study were carried out with a dose of 150 mg/kg BAY-747 in order to minimize the influence of blood pressure lowering on functional outcomes but also have a dose which showed target engagement. 

### 3.3. Plasma Concentration (Pharmacokinetic Effect) of BAY-747

Mice were supplied with food supplemented with 150 mg of BAY-747 per kg of food *ad libitum* for 5 days, following which plasma was collected over various time points ranging from 0 to 24 h to measure the levels of BAY-747. On average each mouse consumed approximately 20 mg/kg of BAY-747, and it was identified that the steady state total plasma concentration (Supplementary Figure S3A) of BAY-747 was 70–270 nM and that of the unbound plasma concentration (Supplementary Figure S3B) was between 8–30 nM, with the levels only slightly higher in the mdx/mTR^G2^ mice compared to C57BL6/J mice. The lowest plasma concentrations were consistently observed at the 10 h timepoint, corresponding to 4 pm in the afternoon. Based on this analysis the exposure levels reached with BAY-747 chow is in the estimated range and in line with the pharmacodynamic effects observed on blood pressure.

## 4. Discussion

DMD is still a rare but devastating disease with a substantially reduced life expectancy. While the efforts for gene therapies for the treatment of DMD are ongoing, the high technical risk associated with high costs involved in the research and development of gene therapeutics for rare diseases could be a limiting factor [25]. Therefore, researchers are still investigating the use of compounds with a known and disease-relevant mode of action, that are already in late-stage development or in the market, which provide the advantages of shorter development times and a well described safety profile. The compound class of sGC stimulators can stimulate the sGC enzyme independent of NO and can thereby increase cGMP levels in muscle cells, which are thought to be beneficial in DMD [7,13]. We therefore investigated in a preclinical “humanized mouse model of Duchenne muscular dystrophy” (mdx/mTR^G2^ mice) [26,27], the effects of the novel sGC stimulator, BAY-747. 

The major findings from this study highlight that, a chronic, 16-week treatment with BAY-747 results in an improvement of skeletal muscle function and damage in mdx/mTR^G2^ mice. Although no improvements were observed in the four-limb hang and overall voluntary running distance, mdx/mTR^G2^ mice that consumed food supplemented with BAY-747 had improved forelimb grip strength and running speed in the voluntary running wheel as well as reduced levels of a key biomarker of skeletal muscle damage, serum creatine kinase. Furthermore, BAY-747 treatment also showed reduced expression of markers of inflammation and fibrosis at the gene expression level but not at the histopathological level. The observed improvements are likely due to the stimulation of the NO/sGC/cGMP pathway by the sGC stimulator since cGMP production in the quadriceps and rectus abdominis muscles of mdx/mTR^G2^ mice were found to be increased after BAY-747 treatment.

In our study, we found that the beneficial effects of treatment of mdx/mTR^G2^ mice with BAY-747 were moderate. This is at least in part in contrast to earlier results in preclinical models with cGMP-increasing drugs like PDE5 inhibitors [10,13]. Sildenafil and tadalafil have shown various therapeutic benefits including reversing cardiac dysfunction [11] and improved respiratory muscle strength and reduced fibrosis of the diaphragm [13]. This enhanced efficacy in these previous studies might be due to the different dosage regimens. Before our treatment study, we performed a pharmacokinetic and pharmacodynamic dose-finding study (Supplementary Results Section) with 50 and 150 mg/kg of BAY-747. The 150-mg/kg dose of BAY-747 had a moderate blood pressure-reducing effect, which indicated target engagement in the resistance vessels of the mice. We decided not to increase the dose further since a significant blood pressure drop could have a potential impact on the functional muscle read outs. To address whether the chosen dose allowed for proper exposure to striated muscle tissues, we quantified the concentration of cGMP in different muscles and found an increase of cGMP content in the quadriceps and the rectus abdominus, suggesting target engagement in these muscle types as well. In contrast, in the diaphragm, the concentration of cGMP remained unchanged after BAY-747 treatment. Since we used whole muscle homogenates for the quantification of cyclic nucleotides, we could not exclude higher local concentrations in the diaphragm due to the high intracellular compartmentalization of the cGMP signaling cascade. Overall, there is only little information on cGMP concentrations in muscles and it therefore would be very interesting to confirm and extend these finding on cNMPs in the future, which will also help to better understand the role of cGMP in striated muscles. Concomitantly, the levels of cAMP were also increased in the rectus abdominus, which could be due to cGMP to cAMP crosstalk via cGMP-induced inhibition of PDE3. 

Additionally, the treatment effects in our study might be also impacted by the time of initiation of the BAY-747 treatment. Early diagnosis and pharmacological intervention are crucial for the treatment and management of symptoms associated with DMD. Boys with DMD are usually diagnosed between the ages of three to five years and treatments and interventions start immediately post-assessment. In our study, treatment was initiated at six weeks of age in mdx/mTR^G2^ mice, and this is perhaps not early enough to show the full potential benefits of BAY-747. In fact, in a recent study that also used the mdx/mTR mouse model of DMD, ifetroban, an antagonist of the thromboxane-prostanoid receptor, treatment was initiated as early as three weeks of age and continued for an additional three months [28].

From a mechanistical perspective, the failure of PDE5 inhibitor drugs might be due to the low NO production in DMD. In addition, we could also not exclude that oxidative stress could at least in part blunt NO as well as sGC stimulator effects. According to previously published data it is evident that DMD is also a condition associated with oxidative stress in the skeletal muscles (Tidball 2018, Grounds 2020). Oxidative stress does not only impair NO binding to the sGC enzyme thereby interrupting NO/sGC/cGMP signaling, but also limits the effects of sGC stimulators like BAY-747. Therefore, another class of sGC modulating compounds known as sGC activators, which activate oxidized and heme free sGC and restore sGC signaling under oxidative stress conditions [29] might be a more effective alternative to sGC stimulators. In the future, head-to-head comparisons of sGC stimulators and sGC activators are needed to fully exploit the treatment opportunities of cGMP increase by sGC agonists in DMD. 

Interestingly, in our study, we observed different degrees of improvement and efficacy in different skeletal muscles such as the quadriceps, rectus abdominis, and diaphragm. Skeletal muscles can be classified according to various categories, for example based on the predominant fiber type, i.e., slow (Type I) or fast (Type II) twitch, type of energy used i.e., aerobic (oxidative) or anaerobic (glycolytic) etc. [30]. The primary mechanism of action of NO-sGC-cGMP modulators in improving exercise efficiency is by causing vasodilatation and thereby improving the amount of oxygen delivered to the muscle fibers, as well as reducing the cost of ATP and oxygen used for muscle contraction and ATP resynthesis [9]. Our running wheel data demonstrated that mdx/mTR^G2^ mice treated with BAY-747 displayed good sprint but not endurance efficiency, as they were able to run the same distance as untreated mdx/mTR^G2^ mice but were able to run at a much faster pace. Therefore, it appears that chronic treatment with BAY-747 plays a more effective role in muscles with fast glycolytic fibers which are able to generate strong contractions but only for a short while as they fatigue much faster when compared to slow oxidative fibers [31]. 

Overall, chronic sGC stimulation with BAY-747 improved various parameters in mdx/mTR^G2^ mice. However, the treatment effects were moderate and dose escalation might not be appropriate due to blood pressure lowering. Therefore, sGC stimulators might provide an adjunctive treatment modality for DMD patients rather than a stand-alone option, similar to current treatment regimens which include combinations like MR-antagonists with ACE-inhibitors [32]. Additional preclinical studies are necessary to substantiate the treatment effects of sGC stimulators in DMD especially when combined with MR-antagonists or ACE-inhibitors. In addition, since DMD is associated with inflammation and increased oxidative stress it might be very interesting to investigate the efficacy of sGC activators as a stand-alone option in mdx mice for evaluation of the treatment potential of this drug class in DMD. 

## 5. Materials and Methods

### 5.1. Animal Model and Experimental Design

All experiments were performed according to the guidelines approved by the local animal welfare authorities for the German state of North-Rhine Westphalia (Landesamt für Natur, Umwelt und Verbraucherschutz Nordrhein-Westfalen; 400a152). 

The mdx^4cv^/mTR^G2^ (hereon referred to as mdx/mTR^G2^) mouse model has previously been described as the “humanized mdx mouse model of DMD” by Blau and colleagues [26,33]. The DMD^mdx4cv^ mice have a nonsense mutation in Exon 53 of the gene encoding for dystrophin, coding for a truncated dystrophin protein, whereas the mTR^−/−^ mice are homozygous for the telomerase RNA component null allele and lack telomerase activity. Male mdx/mTR^G2^ mice approximately 6–7 weeks of age were used for the study reported in this paper. Age-matched C57BL6/JRj (Janvier Labs, Le Genest-Saint-Isle, France) mice were used as wild type controls. The license for the mdx/mTR^G2^ mice was obtained from Stanford University and breeding pairs were obtained from Jackson Laboratories, Bar Harbour, ME, USA (Stock No.: 023535), and the breeding of both the mdx/mTR^G2^ and C57BL6/JRj mice were performed by Janvier Labs. The mice were housed in individual cages under standard conditions including 12-h light/dark cycle, approximately 22 °C temperature and 55% humidity. Standard mouse food was commercially purchased and was provided *ad libitum* as is (untreated) or supplemented with 150 mg of the sGC stimulator BAY-747 (*N*-(2-amino-2-methylbutyl)-8-[(2,6-difluorobenzyl)oxy]-2,6-dimethylimidazo[1,2-a]pyridine-3-carboxamide) per kg of food (Ssniff Spezial-diäten GmbH, Soest, Germany) for a total treatment duration of 16 weeks. Body weight and food consumption was monitored every week. At the end of the study, animals were killed under deep anesthesia and tissue and organ samples were collected for further analysis and histopathological scoring.

### 5.2. Radiotelemetry for Short-Term Monitoring of Blood Pressure and Heart Rate

To examine the effect of BAY-747 on blood pressure and heart rate, radiotelemetry was used. The 20-week-old adult C57BL6/J and mdx/mTR^G2^ mice were implanted with a telemeter probe (Model TA11PA-C10, Data Sciences International, St. Paul, MN, USA) for monitoring blood pressure in conscious mice. After a 10-day recovery period, baseline recordings were obtained over 4 days. Following this, all mice were given food either untreated or supplemented with BAY-747 50 mg/kg or 150 mg/kg for 1 week, and BP was measured in a cross-over study manner with a wash-out period of 1 week (untreated food) in between.

### 5.3. Measuring Plasma Concentrations of BAY-747

Wild type and mdx/mTR^G2^ mice were fed food supplemented with BAY-747 (150 mg/kg) for 5 days, following which plasma was collected at various time points starting at the end of the dark cycle (active/feeding period), for analysis of plasma concentration. BAY-747 concentration was analyzed in the plasma using an LC-system for mass separation (Kinetex 5 µm C18 100 A LC Column 150 × 4.6 mm) coupled to a 4500 Triple Quad Sciex mass analyzer (positive mode; Framingham, MA, USA). A generic internal standard was added to the samples. A 5-point calibration curve and quality control samples were used for relative quantification.

### 5.4. Voluntary Running Wheel

Prior to treatment, mice were placed in individual running wheel cages (built in-house) for one week. Each running wheel was calibrated at least once every week to ensure accurate recording of wheel revolutions. Since the mice are more active in the night period, the data were recorded daily between 3 pm and 9 am the following day. Following one week of voluntary running, mice were randomized into treatment groups based on the overall distance run. In order to observe treatment effects that are not biased by chronic exercise, we used a staggered approach in assessing the effects of the running wheel. To do this, the mice were housed in their regular cages (sedentary) and only moved to the running wheel cages when required. In our study, mice were placed in the running wheel cages during weeks 8 to 10 and weeks 14 to 16 of treatment, and overall distance and average speed run every 2–3 days was calculated during these periods.

### 5.5. Forelimb Grip Strength

Forelimb grip strength was assessed using a force transducer (Sauter GmbH, Balingen, Germany) as described in the Treat-NMD SOP (use of grip strength meter to assess the limb strength of mdx mice—DMD_M.2.2.001). The assessment was performed in an unblinded manner, by the same experienced operator, and always in the same room, with temperature and humidity controlled at the same time. In brief, a grid-like mesh was attached horizontally to the force transducer, which was set to 0 prior to each measurement. The mouse was lifted by its tail and brought close to the mesh allowing the mouse to grab the mesh with its fore paws. The mouse was slowly pulled back until it lets go off the mesh, and the maximum force built up in resistance was recorded by the force transducer. This was repeated 5 times, with a gap of 1 min between each measurement. The average force measured was normalized to body weight. 

### 5.6. Four-Limb Hang

The four-limb hang assessment was performed as described in the Treat-NMD SOP (The use of four limb hanging tests to monitor muscle strength and condition over time—DMD_M.2.1.005). Since we observed that most wild type mice were able to hang for prolonged periods, for this assessment all the mice were allowed to hang for a maximum of 900 s. If they did not succeed in doing so the first time, they were made to repeat the trial a second or third time with a 10-to-15-min resting period between each trial.

### 5.7. Measurement of Serum Creatine Kinase

Whole blood was collected through the inferior vena cava and left to clot at room temperature for 30 min. The samples were then centrifuged at 1000 rcf and 4 °C for 10 min, following which the serum was transferred to a fresh tube and stored at −80 °C, until ready for analysis. Creatine kinase levels in the serum were measured in the automated clinical chemistry analyzer, ADVIA^®^ Chemistry XPT System (Siemens, Munich, Germany) according to the kit’s instruction for the creatine kinase assay (Siemens, Munich, Germany).

### 5.8. Gene Expression Analysis by RT/qPCR 

Total RNA was extracted from the snap-frozen quadricep tissue using the miRNeasy Mini Kit (Qiagen, Hilden, Germany) according to kit instructions. RNA was quantified and controlled for purity (260/280 absorbance ratio ~2.0) using the NanoDrop. A total of 1µg of RNA was reverse transcribed to cDNA using the ImProm-IITM Reverse Transcription System (Promega, Madison, WI, USA) for use as a template for real-time qPCR analysis. The TaqMan primer/probe sequences for the consecutive qPCR are listed in Supplementary Table S1 (sequences were designed by Primer Express 1.5 Software, Applied Biosystems). qPCR was performed with the qPCR Master Mix Plus (Eurogentech; Seraing, Belgium) on a 7900 HT Fast Real-Time PCR System (Applied Biosystems, ThermoFischer Scientific, Waltham, MA, USA). Ct values were determined by Applied Biosystems’s SDS Software (version 2.4), normalized to the housekeeping gene Rpl32. To calculate the fold-change in mRNA expression relative to wild type control samples, the comparative Ct method was used [34]. 

### 5.9. Flow Cytometry

Single-cell suspensions of various skeletal muscles (quadriceps, rectus abdominis and diaphragm) were obtained by digesting the tissues using the Multi Tissue Dissociation Kit 1 (Miltenyi Biotec, Auburn, CA, USA) according to kit instructions. The cells were passed through a 70 µM cell strainer, erythrocytes were lysed using 1× BD Pharm Lyse™ (BD Biosciences, Franklin Lakes, NJ, USA) and washed with autoMACS^®^ Running Buffer (Miltenyi Biotec, USA). Fc receptors were blocked with CD16/CD32 (BD Biosciences, USA) for 10 min. Extracellular surface markers were stained with an antibody cocktail of the Live/Dead stain, APC-Cy™7 anti-mouse CD45 (clone 30-F11, BD Biosciences, USA), APC anti-mouse Ly-6G (clone 1A8, BD Biosciences, USA), PE anti-mouse F4/80 (clone T45-2342, BD Biosciences, USA), FITC anti-mouse CD86 (clone GL-1, BioLegend, San Diego, CA, USA) each at a 1:100 dilution for 30 min on ice. For intracellular staining, the cells were fixed with Leucoperm™ Reagent A 10 min, washed and following the cells were permeabilized with Leucoperm™ reagent B (Bio-Rad, Hercules, CA, USA) containing BV421™ anti-mouse CD206 (clone C068C2, BioLegend, USA) at a 1:100 dilution. The cells were washed and then analyzed on the BD FACSVerse™ Flow Cytometer (BD Biosciences, USA). Data analysis was performed using FlowJo 7.6 software (FlowJo, Ashland, OR, USA) and normalized to tissue weight.

### 5.10. Histopathology

Skeletal muscle tissues (quadriceps, rectus abdominis and diaphragm) were fixed in 10% neutral buffered formalin and processed for histopathology. One cross- and/or longitudinal-section of each the muscle samples were embedded in Paraplast^®^ (Sigma-Aldrich, USA). Paraffin sections, approximately 4 µm thick were prepared and stained with hematoxylin and eosin (H&E) for identification of muscle degeneration/regeneration and fiber necrosis. Sirius Red/Fast Green (SRFG) stain was applied for the detection of collagen deposition. Per mouse, three different muscle samples and two stains per sample were evaluated using the same localization, in total 6 slides/per mouse. The slides were analyzed in an unblinded fashion. Histopathological lesions were graded by a certified veterinary pathologist semi-quantitatively. The overall grading of a certain lesion is based on the evaluation of the entire area of the standardized muscle sample. Thus, the grading takes into account the severity of a single lesions as well as the multiplicity and distribution pattern of this change (i.e., number of affected fibers, affected area, amount of collagen deposition). Two major terms describe a certain muscle lesion: degeneration/necrosis (H&E) and fibrosis (SR_FG). The grading system applied is part of the Pathdata™system which is used for the collection and storage of microscopic data from toxicological and pharmacological studies. For the semiquantitative grading of a certain microscopic change the following scores were applied: GRADE 0 = no finding present; GRADE 1 = minimal/very few/very small; GRADE 2 = slight/few/small; and GRADE 3 = moderate/moderate number/moderate size.

### 5.11. Measurement of cNMPs in Skeletal Muscle Tissues

sGC stimulators are known to increase cGMP levels by binding to the reduced, heme-containing sGC enzyme. To assess whether BAY-747 increased 3′,5′-cyclic guanosine monophosphate (cGMP) levels and had any impact on 3′,5′-cyclic adenosine monophosphate (cAMP) levels, we measured the cyclic nucleotides levels in snap frozen skeletal muscle tissues (quadriceps, rectus abdominis and diaphragm) and heart using liquid chromatography coupled with tandem mass spectrometry (HPLC-MS/MS) using the UFLC HPLC system (Shimadzu, Kyoto, Japan) and the QTRAP5500™ triple quadrupole mass spectrometer (ABSciex, Framingham, MA, USA). A detailed description of the materials and methods used were previously reported [35,36]. 

### 5.12. Statistics

Running wheel data analysis: To summarize the overall distance and average speed data per block across time, the area under the curve (AUC) is calculated for each animal. AUC is log transformed to better fulfil the assumption of normality for the subsequent Dunnett’s test, comparing mdx/mTR^G2^ mice on BAY-747 with untreated wild type and mdx/mTR^G2^ mice, respectively. The family-wise error rate is set to 0.05. All results are transformed back to their original scale. The difference between group averages from the log-scaled data then corresponds to ratios on the original scale. The analysis was carried out using SAS 9.4 and R version 4.0.2.

All other data analysis: Data are expressed as mean ± standard error of mean (SEM). All analyses were perform using GraphPad Prism software v8 (GraphPad Software, San Diego, CA, USA). To identify outliers a ROUT test with Q = 1% was used. To identify statistical differences between groups, a one-way ANOVA was first performed, and if significant this was followed by a post-hoc Dunnett’s multiple comparisons test to identify which groups were different from untreated mdx/mTR^G2^ mice. Similarly, when measurements were repeated over time in the same mouse, a two-way repeated measures ANOVA was performed, followed by a Dunnett’s multiple comparison test. *p* < 0.05 was considered significant.

## Data Availability

All raw data and material is available and documented on file.

## Supplementary Materials

The following are available online at https://www.mdpi.com/article/10.3390/ijms22158016/s1, Figure S1: the body weight and food consumption of mice during the treatment period. (A) Body weight and (B) food consumption measured weekly of the three groups throughout the 16-week treatment period. All values are expressed as mean ± SEM. ns, not significant for two-way ANOVA followed by Dunnett’s multiple comparison post-hoc test, Figure S2: Effects of 50 and 150 ppm (ppm = mg of compound per kg of food) of BAY-747 on blood pressure and heart rate. Treatment with BAY-747 has a dose-dependent effect on systolic blood pressure and heart rate, but no significant effect on diastolic or mean arterial pressure. Effect of consumption of untreated food or food supplemented with BAY-747 at 50 ppm and 150 ppm on (A) systolic blood pressure, (B) heart rate, (C) diastolic blood pressure and (D) mean arterial pressure. All values are expressed as mean ± standard deviation. No statistical analysis was performed, Figure S3: Pharmacokinetic effect (plasma concentrations) of 150 ppm BAY-747. (A) Total and (B) unbound plasma concentration of BAY-747 after consumption of food supplemented with 150 ppm of BAY-747, measured at various time points over a 24-h period. Each point consists of data from *n* = 3 from separate mice. All values are expressed as mean ± standard deviation. No statistical analysis was performed. Table S1: Primer/probe sequence of genes analyzed.

## Abbreviations

µg—microgram: ACTA2—actin alpha 2; BMD—Becker muscular dystrophy; cAMP—cyclic adenosine monophosphate; CCL2—chemokine ligand 2; cDNA—complementary deoxyribonucleic acid; cGMP—cyclic guanosine monophosphate; CK—creatine kinase; COL—collagen; Ct—threshold cycle; CTGF—connective tissue growth factor; DGC—dystrophin glycoprotein complex; DMD—Duchenne muscular dystrophy; H&E—hematoxylin and eosin; ICAM1—intercellular adhesion molecule 1; IL—interleukin; kg—kilogram; LC—liquid chromatography; mg—milligram; MMP—matrix metalloproteinase; NO—nitric oxide; NOS—nitric oxide synthase; NOX—NADPH oxidase; PCR—polymerase chain reaction; PDE—phosophodiesterase; PDEi—phosophodiesterase inhibitor; RNA—ribonucleic acid; RPL32—ribosomal protein L32; SERPINE1—plasminogen activator inhibitor-1; sGC—soluble guanylate cyclase; SPP1—osteopontin; SR/FG—Sirius Red/Fast Green; TGF—transforming growth factor; TIMP—tissue inhibitor of metalloproteinase; TNF—tumor necrosis factor; WT—wild type.

## References

[1] Sun C., Shen L., Zhang Z., Xie X. (2020). Therapeutic Strategies for Duchenne Muscular Dystrophy: An Update. Genes.

[2] Birnkrant D.J., Bushby K., Bann C.M., Alman B.A., Apkon S.D., Blackwell A., Case L.E., Cripe L., Hadjiyannakis S., Olson A.K. (2018). Diagnosis and management of Duchenne muscular dystrophy, part 2: Respiratory, cardiac, bone health, and orthopaedic management. Lancet Neurol..

[3] Birnkrant D.J., Bushby K., Bann C.M., Apkon S.D., Blackwell A., Brumbaugh D., Case L.E., Clemens P.R., Hadjiyannakis S., Pandya S. (2018). Diagnosis and management of Duchenne muscular dystrophy, part 1: Diagnosis, and neuromuscular, rehabilitation, endocrine, and gastrointestinal and nutritional management. Lancet Neurol..

[4] Birnkrant D.J., Bushby K., Bann C.M., Apkon S.D., Blackwell A., Colvin M.K., Cripe L., Herron A.R., Kennedy A., Kinnett K. (2018). Diagnosis and management of Duchenne muscular dystrophy, part 3: Primary care, emergency management, psychosocial care, and transitions of care across the lifespan. Lancet Neurol..

[5] Loboda A., Dulak J. (2020). Muscle and cardiac therapeutic strategies for Duchenne muscular dystrophy: Past, present, and future. Pharmacol. Rep..

[6] Dombernowsky N.W., Olmestig J.N.E., Witting N., Kruuse C. (2018). Role of neuronal nitric oxide synthase (nNOS) in Duchenne and Becker muscular dystrophies—Still a possible treatment modality?. Neuromuscul. Disord..

[7] Moon Y., Balke J.E., Madorma D., Siegel M.P., Knowels G., Brouckaert P., Buys E.S., Marcinek D.J., Percival J.M. (2017). Nitric Oxide Regulates Skeletal Muscle Fatigue, Fiber Type, Microtubule Organization, and Mitochondrial ATP Synthesis Efficiency Through cGMP-Dependent Mechanisms. Antioxid. Redox Signal..

[8] Sander M., Chavoshan B., Harris S.A., Iannaccone S.T., Stull J.T., Thomas G.D., Victor R.G. (2000). Functional muscle ischemia in neuronal nitric oxide synthase-deficient skeletal muscle of children with Duchenne muscular dystrophy. Proc. Natl. Acad. Sci. USA.

[9] Jones A.M., Thompson C., Wylie L.J., Vanhatalo A. (2018). Dietary Nitrate and Physical Performance. Annu. Rev. Nutr..

[10] Asai A., Sahani N., Kaneki M., Ouchi Y., Martyn J.A., Yasuhara S.E. (2007). Primary role of functional ischemia, quantitative evidence for the two-hit mechanism, and phosphodiesterase-5 inhibitor therapy in mouse muscular dystrophy. PLoS ONE.

[11] Adamo C.M., Dai D.F., Percival J.M., Minami E., Willis M.S., Patrucco E., Froehner S.C., Beavo J.A. (2010). Sildenafil reverses cardiac dysfunction in the mdx mouse model of Duchenne muscular dystrophy. Proc. Natl. Acad. Sci. USA.

[12] Hammers D.W., Sleeper M.M., Forbes S.C., Shima A., Walter G.A., Sweeney H.L. (2016). Tadalafil Treatment Delays the Onset of Cardiomyopathy in Dystrophin-Deficient Hearts. J. Am. Heart Assoc..

[13] Percival J.M., Whitehead N.P., Adams M.E., Adamo C.M., Beavo J.A., Froehner S.C. (2012). Sildenafil reduces respiratory muscle weakness and fibrosis in the mdx mouse model of Duchenne muscular dystrophy. J. Pathol..

[14] Nelson M.D., Rader F., Tang X., Tavyev J., Nelson S.F., Miceli M.C., Elashoff R.M., Sweeney H.L., Victor R.G. (2014). PDE5 inhibition alleviates functional muscle ischemia in boys with Duchenne muscular dystrophy. Neurology.

[15] Martin E.A., Barresi R., Byrne B.J., Tsimerinov E.I., Scott B.L., Walker A.E., Gurudevan S.V., Anene F., Elashoff R.M., Thomas G.D. (2012). Tadalafil alleviates muscle ischemia in patients with Becker muscular dystrophy. Sci. Transl. Med..

[16] Victor R.G., Sweeney H.L., Finkel R., McDonald C.M., Byrne B., Eagle M., Goemans N., Vandenborne K., Dubrovsky A.L., Topaloglu H. (2017). A phase 3 randomized placebo-controlled trial of tadalafil for Duchenne muscular dystrophy. Neurology.

[17] Stasch J.P., Becker E.M., Alonso-Alija C., Apeler H., Dembowsky K., Feurer A., Gerzer R., Minuth T., Perzborn E., Pleiss U. (2001). NO-independent regulatory site on soluble guanylate cyclase. Nature.

[18] Stasch J.P., Schmidt P., Alonso-Alija C., Apeler H., Dembowsky K., Haerter M., Heil M., Minuth T., Perzborn E., Pleiss U. (2002). NO- and haem-independent activation of soluble guanylyl cyclase: Molecular basis and cardiovascular implications of a new pharmacological principle. Br. J. Pharmacol..

[19] Mittendorf J., Weigand S., Alonso-Alija C., Bischoff E., Feurer A., Gerisch M., Kern A., Knorr A., Lang D., Muenter K. (2009). Discovery of riociguat (BAY 63-2521): A potent, oral stimulator of soluble guanylate cyclase for the treatment of pulmonary hypertension. ChemMedChem.

[20] Stasch J.P., Evgenov O.V. (2013). Soluble guanylate cyclase stimulators in pulmonary hypertension. Handb. Exp. Pharmacol..

[21] Tidball J.G., Wehling-Henricks M. (2014). Nitric oxide synthase deficiency and the pathophysiology of muscular dystrophy. J. Physiol..

[22] Kobayashi Y.M., Rader E.P., Crawford R.W., Iyengar N.K., Thedens D.R., Faulkner J.A., Parikh S.V., Weiss R.M., Chamberlain J.S., Moore S.A. (2008). Sarcolemma-localized nNOS is required to maintain activity after mild exercise. Nature.

[23] Brancaccio P., Maffulli N., Limongelli F.M. (2007). Creatine kinase monitoring in sport medicine. Br. Med. Bull..

[24] Follmann M., Ackerstaff J., Redlich G., Wunder F., Lang D., Kern A., Fey P., Griebenow N., Kroh W., Becker-Pelster E.M. (2017). Discovery of the Soluble Guanylate Cyclase Stimulator Vericiguat (BAY 1021189) for the Treatment of Chronic Heart Failure. J. Med. Chem..

[25] Vitiello L., Tibaudo L., Pegoraro E., Bello L., Canton M. (2019). Teaching an Old Molecule New Tricks: Drug Repositioning for Duchenne Muscular Dystrophy. Int. J. Mol. Sci..

[26] Mourkioti F., Kustan J., Kraft P., Day J.W., Zhao M.M., Kost-Alimova M., Protopopov A., DePinho R.A., Bernstein D., Meeker A.K. (2013). Role of telomere dysfunction in cardiac failure in Duchenne muscular dystrophy. Nat. Cell Biol..

[27] Yucel N., Chang A.C., Day J.W., Rosenthal N., Blau H.M. (2018). Humanizing the mdx mouse model of DMD: The long and the short of it. NPJ Regen. Med..

[28] West J.D., Galindo C.L., Kim K., Shin J.J., Atkinson J.B., Macias-Perez I., Pavliv L., Knollmann B.C., Soslow J.H., Markham L.W. (2019). Antagonism of the Thromboxane-Prostanoid Receptor as a Potential Therapy for Cardiomyopathy of Muscular Dystrophy. J. Am. Heart Assoc..

[29] Hahn M.G., Lampe T., El Sheikh S., Griebenow N., Woltering E., Schlemmer K., Dietz L., Gerisch M., Wunder F., Becker-Pelster E. (2021). Discovery of the Soluble Guanylate Cyclase Activator Runcaciguat (BAY 1101042). J. Med. Chem..

[30] Scott W., Stevens J., Binder-Macleod S.A. (2001). Human skeletal muscle fiber type classifications. Phys. Ther..

[31] Radák Z. (2018). Skeletal Muscle, Function, and Muscle Fiber Types. The Physiology of Physical Training.

[32] Rafael-Fortney J.A., Chimanji N.S., Schill K.E., Martin C.D., Murray J.D., Ganguly R., Stangland J.E., Tran T., Xu Y., Canan B.D. (2011). Early treatment with lisinopril and spironolactone preserves cardiac and skeletal muscle in Duchenne muscular dystrophy mice. Circulation.

[33] Sacco A., Mourkioti F., Tran R., Choi J., Llewellyn M., Kraft P., Shkreli M., Delp S., Pomerantz J.H., Artandi S.E. (2010). Short telomeres and stem cell exhaustion model Duchenne muscular dystrophy in mdx/mTR mice. Cell.

[34] Schmittgen T.D., Livak K.J. (2008). Analyzing real-time PCR data by the comparative C(T) method. Nat. Protoc..

[35] Bahre H., Danker K.Y., Stasch J.P., Kaever V., Seifert R. (2014). Nucleotidyl cyclase activity of soluble guanylyl cyclase in intact cells. Biochem. Biophys. Res. Commun..

[36] Monzel M., Kuhn M., Bahre H., Seifert R., Schneider E.H. (2014). PDE7A1 hydrolyzes cCMP. FEBS Lett..

