# Supplementary material for: Assessing the Use of the sGC Stimulator BAY-747, as a Potential Treatment for Duchenne Muscular Dystrophy
Source: Int J Mol Sci. 2021 Jul 27;22(15):8016. doi: 10.3390/ijms22158016 (PMC8347633; doi:10.3390/ijms22158016)
Supplement: Supplementary file 1 [file ijms-22-08016-s001.zip › ijms-1222886-supplementary table.pdf]

International Journal of
Molecular Sciences

MDPI

Supplementary Methods

Supplementary Table S1: Primer/probe sequence of genes analyzed

Target Gene	Forward	Reverse	Probe
NOS1	TATGTACAGGACGTGCTGCA	GCCTCCTTGCTCCTTCAGG	CAGCTGGCCGAGTCTGTGTAC-CGC
NOS2	TTGCTCCCTTCCGAAGTTTCT	CCTCCTTTGAGCCCTTTGTG	CAGCGGCTCCATGACTCCCA
NOS3	CTGGATGAAGCCGGTGAC	GCAATGTGAG-TCCGAAAATG	CTGCGGGATCAGCAACGCTACC
NOX1	GTGGGGGTTTTCTTATGTGG	CCAGACTCGAG-TATCGCTGA	ACTTTGGCAAAAAGCCTGCG-CAAAC
NOX2	CACCACAATAGGCGTTTTCC	CGGATTCTGAGTTGGA-GATGC	CCCTGAAGCCTT-GGCTGAAACTCTCA
NOX4	ATTGGGCGTCCTCGGTGGAA	TGGGTCCACAG-CAGAAAACTC	ATAGCAAAATGTAACAGAGGGA AAACAGT
GUCY1A1	CGTCTTTTAGAGATGGAGA-TAGAGCTT	TCACACCGCTTGGGTTA-GAACTA	TGACAAAGCACTTGTCTAG-CATTTAGGCAGTCC
GUCA1A2	CCAGACAACTTTCCGAAGGA	GGCTTTGGGTCAG-TCCTTAAC	TTCCTGGGGTCTGC-TATTTCCTGGA
GUCY1B1	TCTGTT-GCCAATGAGCTGAGA	AGGATGGTCACATTGTCG-TATCTTT	CAAGCGCCCAGTGCCTGCC
GUCY1B2	CAGGTGTTGTGGGAGACAAG	TCCTA-GAGGCCGTGTTTACG	CCCGGTACTGCCTGTTT-GGTGACAC
PRKG1	GAGGGCTTTAATTGGGAAGG	TGCTTGTGTCTGTGGGTGA	CACCTCCCATAATTCCAAGTGTT-GCG
PRKG2	TGGCCGAGGGTAGACTTGAG	AATAGCCAG-TTCCCCAAACGT	CCAGGGAGAGAAGTTGC-TATCATCCATCC
PDE1a	GACGAGGTCGGACGTTGCT	CTGCGCTGACGTGGTGATT	TTCTGTACAACGACCGCTCAG-TGCTTGA
PDE1b	GACTCGG-CATAGCCTCATCAG	ATAGCCTGTCTCCAAGGCC TC	CGCTTTAAGATTCCCACAG-TGTTTCTGATGA
PDE1c	CCACCGGCTGTAATCGATG	TGGCCTCATT-GAGGGAAAAG	CGAAGGACCAC-GTATCCACATCCTTCA
PDE2a	ATCCTGAACATCCCAGATGC	AGGGAAGCAGA-GAATGTTGC	CCCATCCGCTTTTCTATCGCGG
PDE3a	AGAGCAGATCCAGGC-TATCAA	CCAGGGTCTCCTCTGCTCTT	AGGAAGAGGAAGAGAAGGG-GAAGCC
PDE3b	TGGATCGTTCTTCTCCTCAA	CAGCATCATAGGAGTT-GCACA	CTTTTATCACCCACATT-GTGGGCCC
PDE4a	CAAATGGGGCCTGAACAT	ACATGATACAGCTGAG-TGAACG	TTTGTGTGTCGGAGTACGCTG-GAGG
PDE4b	CAACGCCAGACACTCAG-GAA	GGAGGCTCATGTGTTT-GGACA	TGGTGATTGACATGGTGTTGG-CAACTG
PDE4c	CCACACCAGCCTTCCCACAG	CTGCTCCTCCTGATCTGTCT	ATCCCACGCTTTGGGGTCC
PDE4d	CTCGGAACTCGCTCTGATGT	ACTTAAAGCCCAC-CGCCAAG	CAACGACTCCTCGGTCCTAGA-GAACCATCA
PDE5a	CCGACTTCAGCTTCAG-TGACTT	GGTCAG-TGAACATCCGAATTG	TGTCTGATCTG-GAAACAGCGCTGTG

PDE6a	CATGCTGGATGGGATCACTA	GCCTTCATCTTGGCTTCGTA	AAGGAATGGAAGGCGCTGGCTGAT
PDE6b	TCGATTTCACGAAGAGATCC	TCGTCAGCTAG-TGCTTTCCA	CCCATGTTTGACCGACTG-CAAAATA
PDE6c	CAGGCTGCTGAACGTCAC	CCAGCCAAC-TATCCCAATGT	AGGACAACCTCGTGGCCCCTGA
PDE6d	CGAGTTTGGCTTTGTGATCC	ACTCAGGCGCTGCTTC-TATC	ACTCCACAAACACCTGGCAG-TCCTT
PDE6h	AGCGTTCAGCCATCTGGA	AGCCTCTGGGCTCCTCATA	CATGAGCTTGCTCAGTTCGG-GATCA
PDE7a	TGGAGGCTCAGATAGGTGCT	CCAAGTGA-GATCTAAACAATGACA	CACGGATATCAGTCGCCAGAAC-GAG
PDE7b	CTGGGCTCCCTCATCTTG	TCTTTATTGTG-GAGGTGAGCTTT	CAGACAGAATGAG-TTTCTGACCCGC
PDE8a	TCGTTGACCTGCCTAACCTT	TTCGAA-GCTTCTTCTCATCCA	TGCAGCACCTA-GATGACAACTTCAGG
PDE8b	CTGGATGACCTCAAGTGCAA	TCACAGTGTCCCCTTCTCG	CTTCCATCTGACAGCTGAA-GCCACG
PDE9a	CATGGACCGAGA-CAAAGTGA	TGGGATCAG-GACAAACTTGA	AAGCAACAGCCCAAATT-GGGTTCA
PDE10a	GAGCAGGTGCTGGAGATCAT	TCCTCCAACTGCTTCCTGTT	CGCAAAGCCATCATCGCCAC
PDE11a	TTTTCCCTTGATGTTGATGC	TTCTGTAC-CATCCCCAGCTC	TCACAGCCGCTCTAC-GGATGTTCAT
TNF	TCTCAAAATTCGAG-TGACAAGC	CAGCCACTCCAGCTGCTC	CACGTCGTAGCAAACCAC-CAAGTG
SPP1	ATTTGCTTTTGCCTGTTTG	CTCCTCTGAGCTGCCAGAAT	CTCCTCCCTCCCGGTGAAAGTGACT
IL-1β	GACCTTCCAGGATGAGGACA	TGAGTCACAGAG-GATGGGCT	AGCACCTTCTTTTCCTTCATCTTT-GAAGAA
IL-6	CGGACACTGGCTGTGGAATT	GCCAGGATCTTGGGCAACT	AAATGATGTCTGAAGTTATTGCTGCC
CCL2	AAGAGGATCACCAGCAG-CAG	TTCTTGGGGTCAGCACAGA	TCCCAAAGAAGCTGTAGTTTTT-GTCACCA
ICAM1	AACCAGACCCTGGAACTGC	AGGTCCAGTTCCCCAAGC	TGCTGTATGGTCCTCGGCTGGA
COL1A1	GACCTCAAGATGTGCCACTC	AGGTTGCAGCCTT-GGTTAGG	ACTGGAAGAGCGGAGAGTACTG-GATC
COL1A2	TGCAGGGTTCCAACGATGTT	CGACTAGGACAGAG-TAGGTGAACCT	AACTTGTTGCTGAGGGC
COL3A1	CTGGTGAGCGAGGAC-GACCA	GAATCCTGCAGTTCCAG-GAGG	CTCGAGGCAATGATGGTGCTCGGGGCAG
TGFβ1	TGAGTGGCTGTCTTTTGACG	AGCCCTGTATTCCGTCTCCT	ACTGGAGTTGTACGGCAG-TGGCTGA
TGFβ2	ACCTTTTTGCTCCTGCATCTG	GTCGAGGGTGCTGCAGGTA	TCCCGGTGGCGCTCAGTCTGT
TGFβ3	CCCTATCAGGTCCTGGCACTT	CCTCTCCCCGTGCATCTCT	AACAGCACCCGGGAGTTGCTGG
MMP2	CTATGTCCACTGTGGGTG-GAAA	TTGTTGCCCAG-GAAAGTGAAG	CAGAAGGTGCCCCATGTGTCTTC
MMP9	CCTGGAACTCACACGACATC	GAAACTCACAC-GCCAGAAGA	CCAAGACAAAGCC-TATTTCTGCCATGG
TIMP1	TGCCTGCCACGGAATCCAGG	TCCTGGGG-GAAGGCTTCAGGT	TGCACCTGGAGATCCCTT-GGGGCCCG

TIMP3	CCCCAGGATGCCTTCTG-CAAC	CCTCCTTCAC-CAGCTTCTTTC	CCGACATCGTGATCCGGGCCAAA
ACTA2	TTGCTGACAGGATGCAGAAG	GTTCTGGAGGGGCAATGAT	CTCGCACCCAGCACCATGAAGA
Fibronectin	GTCATTGCCCTGAAGAACAA	AACCAGTTGGGGAA-GCTCAT	AAGAGTGAGCCCCTGATTGG-GAGG
CTGF	CCGCCAACCGCAAGATC	CACCGACCCACCGAAGAC	TGTGCACTGCCAAAGATGGTG-CAC
SERPINE	CCCCACGGAGATGGTTATAG	ATCACTTGGCCCATGAA-GAG	CGGCACAACCCGACAGAGACAA
RPL32	ACTGGAG-GAAACCCAGAGGC	CATCAG-GATCTGGCCCTTGA	TCGACAACAGGGTGCGGAGAAGG
