# Supplementary figures and images for: Assessing the Use of the sGC Stimulator BAY-747, as a Potential Treatment for Duchenne Muscular Dystrophy
Source: Int J Mol Sci. 2021 Jul 27;22(15):8016. doi: 10.3390/ijms22158016 (PMC8347633; doi:10.3390/ijms22158016)

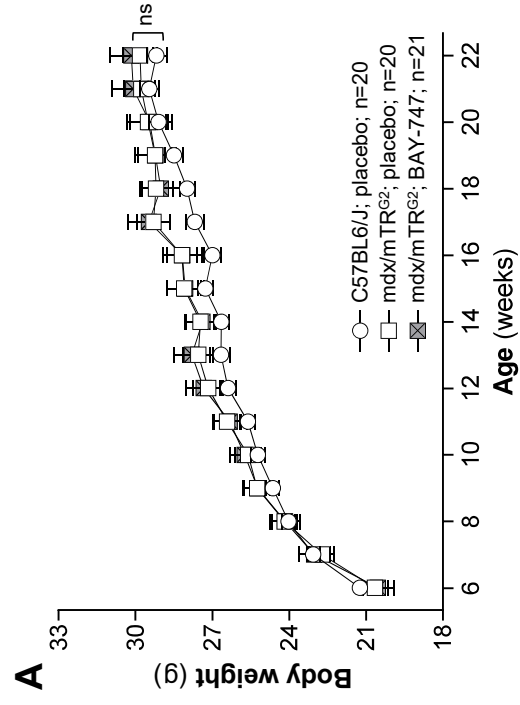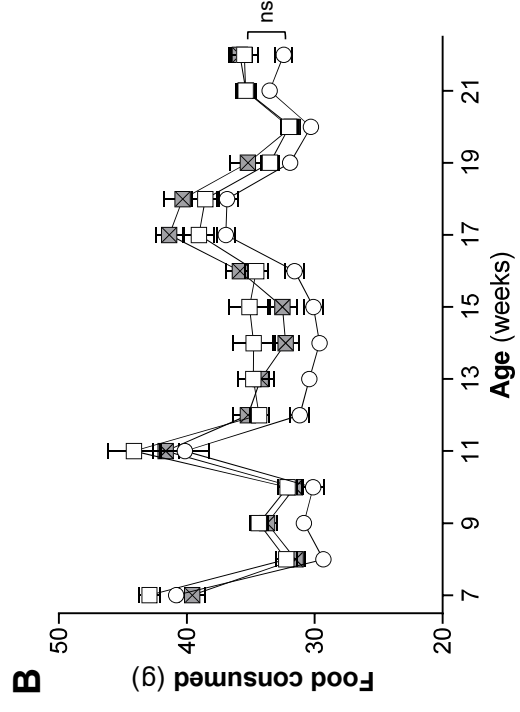

Supplementary Figure S1



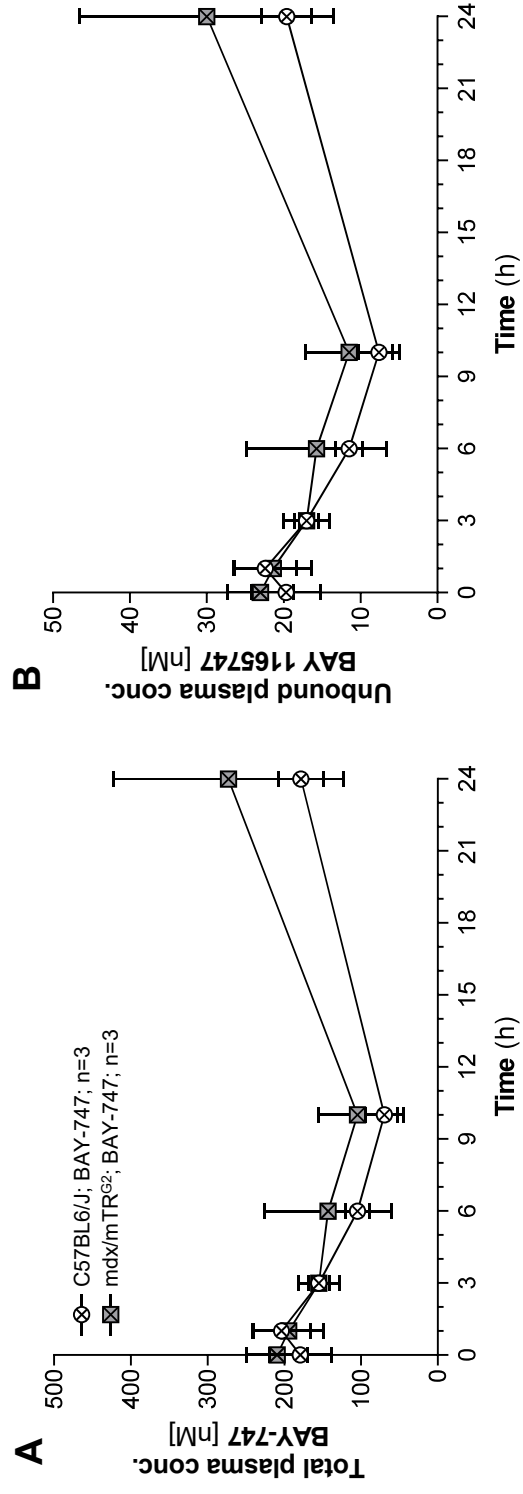

Supplementary Figure S3

Supplement: Supplementary file 1 [file ijms-22-08016-s001.zip › ijms-1222886-supplementary figures.pdf]
